# Traditional, complementary and integrative health within universal systems: a transdisciplinary integrative approach to chronic conditions in the Brazilian SUS

**DOI:** 10.3389/fpubh.2026.1757824

**Published:** 2026-03-26

**Authors:** Frederico M. Cohrs, Fernando A. C. Bignardi

**Affiliations:** 1 Universidade Federal de São Paulo–Escola Paulista de Enfermagem, São Paulo, Brazil; 2 CABSIN (Brazilian Academic Consortium for Integrative Health), Health and Nature Committee and Medical Rationalities Committee, São Paulo, Brazil

**Keywords:** Brazilian Unified Health System (SUS), chronic conditions, chronic disease, chronic illness, epidemiological segmentation, planetary health, sustainable development, traditional complementary and integrative health (TCIH)

## Abstract

In universal health systems such as the Brazilian Unified Health System (SUS), chronic conditions and multimorbidity threaten the sustainability of care. This Perspective connects Traditional, Complementary and Integrative Health (TCIH) with a Transdisciplinary Integrative Health Approach to reframe chronic conditions. Grounded in three pillars (complexity, a multidimensional conception of the human being, and the logic of the included third) the approach understands health and disease as emergent properties of living systems integrating physical, metabolic, vital, mental and supramental (spiritual and systemic) dimensions. We summarize limitations of linear risk-factor models, describe how TCIH practices can act at different dimensions and levels of ‘downward causation', and present arterial hypertension with obstructive sleep apnoea as an illustrative case from the Brazilian SUS, including a vignette involving continuous positive airway pressure (CPAP) and multidimensional transdisciplinary diagnosis. Rather than opposing the biomedical model, the framework positions conventional care, TCIH practices and community-based, nature-based interventions as complementary resources within universal systems. We conclude with implications for training family health teams, designing lines of care and using epidemiological segmentation to plan responses centered on the person and the territory that are clinically effective, culturally resonant and ecologically responsible. By integrating TCIH with a transdisciplinary perspective, chronic conditions can be addressed in ways that support both individual wellbeing and the broader agenda of sustainable and equitable universal health systems.

## 1 Introduction

Chronic conditions are a defining feature of contemporary health systems, particularly in countries that have built universal coverage such as the Brazilian Unified Health System (SUS). Non-communicable diseases, long-term mental health problems, chronic pain and complex multimorbidity patterns shape demands on services and professional work (1, 2). Despite advances in prevention and treatment, the dominant framing of chronic conditions remains rooted in a fragmented biomedical paradigm that privileges organs, risk factors and measurable behaviors. Disease tends to be treated as a static label attached to individuals rather than as a dynamic process that unfolds over time and within relationships.

A simple interpretive reading of the English term “disease” as “dis-ease”—a loss of ease or flow–helps to open a different view. Chronic conditions can be understood as situations in which the flow of life across different dimensions no longer unfolds as it could. This resonates with traditional medical systems that do not conceptualize “chronic disease” primarily as a fixed incurable state, but as a long-term pattern that can still be transformed through changes in lifestyle, relationships and meaning. Contemporary lifestyle medicine adopts a similar stance by using structured lifestyle interventions to prevent, treat and, in some cases, induce remission of chronic conditions.

Chronic care models, integrated care strategies and person-centered approaches have improved responses to long-term conditions (3). Yet most frameworks remain strongly influenced by a linear risk-factor logic: risk factors lead to disease, and standardized interventions are expected to correct physiological and behavioral deviations. Multimorbidity, syndemics, existential suffering and spiritual dimensions are frequently treated as secondary complications rather than as integral aspects of illness.

At the same time, a broader global crisis is evident. Environmental degradation, social inequalities and erosion of meaning point to a disconnection between human societies and Nature. Health patterns, including the rise and persistence of chronic conditions, cannot be separated from this crisis (4). Bodies that accumulate stress, inflammation and exhaustion mirror the tensions of a world marked by acceleration, consumerism and disrupted ecological bonds. Yet predominant frameworks rarely make this connection explicit.

Recent global initiatives, such as the WHO Traditional Medicine Strategy and the WHO Traditional Medicine Global Summit culminating in the Gujarat Declaration, have emphasized the need to integrate traditional, complementary and integrative health into universal health coverage and sustainable development agendas (5, 6). In parallel, the 2030 Agenda for Sustainable Development calls for new approaches that connect clinical effectiveness, equity and ecological responsibility. By proposing ways to integrate TCIH into universal health systems such as the Brazilian SUS, this Perspective speaks directly to Sustainable Development Goal (SDG) 3, particularly targets 3.4 (reducing premature mortality from non-communicable diseases) and 3.8 (achieving universal health coverage). The emphasis on culturally sensitive, territory-based care and on training family health teams is also aligned with SDG 10 on reducing inequalities and SDG 4 on quality education for health professionals.

Transdisciplinarity and integrative health have emerged as avenues to respond to this complexity. However, they are often invoked in a generic way, without a clear conceptual and methodological framework that can guide their application to chronic conditions. This Perspective proposes such a framework: a Transdisciplinary Integrative Health Approach grounded in three interrelated pillars (complexity, a multidimensional conception of the human being, and the logic of the included third) that can be applied in clinical practice, education and policy. A quantum-inspired multidimensional model is used as a heuristic to illustrate these ideas; it is employed metaphorically, not as a formal physical theory.

Despite growing global interest in integrating TCIH into universal health systems (5, 6), this integration faces substantial challenges and uncertainties. Evidence for many TCIH practices remains limited or of low quality, with heterogeneous outcomes across different conditions and contexts (7, 8). Regulatory frameworks are often insufficient to ensure safety, quality and ethical practice, particularly when traditional knowledge is commodified or decontextualised (9). There are risks of reinforcing inequalities if TCIH becomes a “second-class” option for vulnerable populations while high-technology interventions remain reserved for those with greater resources (10). Furthermore, uncritical integration may fragment care rather than strengthen it, especially when TCIH practitioners and biomedical professionals work in parallel without genuine dialogue or shared decision-making (11).

Existing public health frameworks have addressed chronic conditions from different angles. The biopsychosocial model (12) expanded biomedical understanding by recognizing psychological and social dimensions, yet often remains centered on individual-level interventions. The Chronic Care Model (3) introduced systems thinking and team-based care, but predominantly within a biomedical-managerial logic that may not fully accommodate epistemological plurality. Social determinants of health approaches (13) emphasize structural and political-economic factors, though they may underemphasise subjective experience, meaning-making and spiritual dimensions. Syndemic theory (14) highlights how diseases cluster and interact in contexts of social inequality, but focuses primarily on epidemiological patterns rather than therapeutic integration. The planetary health framework (4) connects human health with ecological integrity, opening space for nature-based and community-based interventions but requiring clearer operationalization within clinical practice and health services organization. Each of these frameworks offers valuable insights; the transdisciplinary integrative health approach proposed here seeks to complement them by explicitly integrating TCIH, articulating multiple dimensions of human experience, and operationalizing the logic of the included third as a methodological bridge.

The objective of this Perspective article is to propose a Transdisciplinary Integrative Health Approach grounded in three interrelated pillars (complexity, a multidimensional conception of the human being (integrating physical, metabolic, vital, mental and supramental (spiritual and systemic) dimensions), and the logic of the included third) as a conceptual and methodological framework for reframing chronic conditions within universal health systems. Using the Brazilian SUS as the primary reference context, we illustrate how this approach can inform clinical practice, interprofessional teamwork, health professions education, service organization and public policy. Rather than presenting empirical data or systematic evidence synthesis, this Perspective offers a normative and heuristic proposal intended to stimulate debate, guide future research and experimentation, and contribute to operationalizing global agendas on TCIH integration (5, 6) and sustainable development (SDG 3, SDG 4, SDG 10) in the context of chronic care. The framework positions conventional biomedical care, TCIH practices and community-based, nature-based interventions as complementary resources that can be planned and delivered together, provided that safety, equity and epistemological rigor are maintained.

### 1.1 Experiences of TCIH integration in universal health systems: lessons from the Brazilian SUS

Brazil's National Policy on Integrative and Complementary Practices (PNPIC), established in 2006 and progressively expanded, represents one of the most comprehensive institutional efforts to integrate TCIH into a universal public health system (15, 16). The policy currently encompasses 29 practices, including acupuncture, herbal medicine, homeopathy, traditional indigenous medicine, community therapy, meditation, and yoga, among others. These are offered primarily through primary health care, with significant variation across municipalities.

Empirical studies and institutional evaluations reveal both achievements and persistent challenges. On the positive side, PNPIC has increased access to TCIH for vulnerable populations who would otherwise not afford private services, promoted cultural recognition of diverse healing traditions, and fostered more person-centered and relationally rich clinical encounters (17, 18). Some municipalities have reported reduced use of medications and emergency services among users of integrative practices, though robust comparative effectiveness studies remain scarce (19). Community therapy circles, in particular, have demonstrated capacity to strengthen social support networks and territorial belonging, addressing dimensions of chronic suffering that conventional biomedical care often overlooks (20).

However, implementation remains uneven and faces structural barriers. Many family health teams lack training in TCIH, leading to referrals to specialists rather than integration into everyday primary care practice (21). Fragmentation persists when TCIH practitioners work in separate clinics or schedules without shared care plans or interprofessional dialogue (11). Regulation and quality assurance are inconsistent, with concerns about safety, informed consent and potential exploitation of traditional knowledge (9). Furthermore, TCIH services are often the first to be cut during budget crises, reflecting their peripheral status within health system priorities (22).

International experiences echo similar patterns. The United Kingdom's National Health Service, for example, has supported acupuncture and osteopathy in some regions, but integration remains patchy and dependent on local champions rather than systemic policy (23). In China, Traditional Chinese Medicine coexists with biomedicine in a dual system that sometimes integrates but often runs parallel, raising questions about genuine transdisciplinarity vs. institutional cohabitation (24). These experiences underscore the need for frameworks that go beyond adding TCIH as an optional complement and instead rethink chronic care models from a transdisciplinary, multidimensional perspective—precisely what this Perspective proposes.

While the integration of TCIH into universal health systems has gained momentum globally, critical appraisal of the evidence base reveals persistent methodological challenges and quality concerns that warrant explicit acknowledgment. Systematic reviews of complementary and alternative medicine research consistently identify inadequate protocol registration, unclear inclusion criteria, incomplete risk of bias assessment, and small sample sizes as prevalent limitations that compromise the reliability of findings (8, 25). Meta-research specifically examining traditional, complementary and integrative medicine demonstrates that evidence quality frequently rates as low or very low using GRADE criteria, with inconclusive or inconsistent results across multiple modalities (26). These methodological weaknesses stem partly from inherent challenges in applying conventional randomized controlled trial designs to complex, individualized, and context-dependent interventions characteristic of many TCIH practices.

Beyond methodological concerns, integration efforts face substantial barriers at multiple levels. Systematic reviews identify financial constraints (particularly lack of insurance coverage), skepticism from conventional practitioners, divergent philosophical outlooks between biomedical and holistic paradigms, and limited formalized institutional relationships as key obstacles to effective integration (27). Epistemological tensions between whole medical systems and conventional biomedicine reflect fundamentally different approaches to knowledge generation, disease conceptualization, and therapeutic intervention, requiring careful negotiation rather than simple addition of practices (28). Regional variations compound these challenges: while TCIH research output has grown rapidly in some regions, methodological and reporting quality remains variable, with adherence to established guidelines inconsistent across contexts (8, 26).

These critical perspectives do not invalidate integrative approaches but underscore the necessity for frameworks that explicitly address methodological rigor, epistemological plurality, and implementation complexity. The transdisciplinary integrative health approach proposed in this Perspective engages these concerns by positioning different knowledge systems as complementary rather than competing, while emphasizing the need for safety, evidence, and equity in integration efforts. Rather than claiming superiority over existing models, we offer this framework as one among several possible approaches to navigate the challenges and opportunities of TCIH integration within universal systems.

A central innovation of the transdisciplinary integrative health approach is the concept of a supradisciplinary diagnosis: a phenomenologically grounded method through which an interprofessional care team constructs a unified, emergent clinical portrait of the person seeking help. Rather than juxtaposing parallel disciplinary assessments, this diagnostic process brings together practitioners from diverse backgrounds (each with their disciplinary perspectives and idiosyncrasies) into a shared perceptual consensus that transcends what any single discipline can achieve alone. Guided by phenomenological observation and aligned by the principle of downward causation, the supradisciplinary diagnosis enables the team to identify how disturbances cascade across multiple dimensions of the person's experience and to design integrative therapeutic responses accordingly (29, 30). This concept is further developed and illustrated through a clinical vignette in Section 3.3.

## 2 Conceptual foundations of the transdisciplinary integrative health approach

### 2.1 Complexity and the multidimensional human being

The first pillar is complexity. Individuals, families, communities and health services are seen as interconnected living systems in which small changes may have disproportionate effects and outcomes cannot be fully predicted from initial conditions. Chronic conditions emerge over time from the interaction of biological processes, life histories, relationships, institutions and environments, rather than from isolated risk factors. Causation is non-linear, recursive and often circular.

The second pillar is a multidimensional conception of the human being. We distinguish five interrelated dimensions. The physical dimension refers to structure, organs and systems, accessed through clinical examination and tests. The metabolic dimension includes the “biological terrain” that connects systems—biochemical regulation, inflammation, energetic balance and microbiota, often explored through dietary histories and laboratory markers. The vital dimension encompasses rhythms of life (sleep–wake cycles, breathing, pulse, daily routines) and subjective vitality. The mental dimension comprises posture, attitudes, personality traits, thoughts and emotions, assessed through structured instruments and biographical narratives. Finally, the supramental dimension (encompassing spiritual and systemic aspects) refers to transpersonal awareness and contemplative experience (the spiritual aspect) as well as biographical configurations and the sense of participation in larger patterns of meaning and destiny (the systemic aspect) (31, 32). Chronic conditions are seen as patterns that simultaneously affect and are affected by all five dimensions.

TCIH practices can potentially act on different combinations of these dimensions. Meditation and contemplative exercises, for example, may influence supramental and mental dimensions while also changing vital and metabolic regulation. Body-based therapies may affect physical and vital layers while opening spaces for emotional processing. The strength and quality of evidence for specific practices vary considerably; our aim in this Perspective is not to provide a systematic review, but to offer a conceptual framework that can guide future evaluative research and policy experimentation in line with global strategies on traditional and complementary medicine (5, 6).

### 2.2 The logic of the included third

The third pillar is the logic of the included third, a principle derived from complexity theory and transdisciplinary epistemology (33, 34) that operates simultaneously at three levels: as an epistemological principle, a methodological tool, and a clinical decision criterion.

As an epistemological principle, the logic of the included third challenges rigid binary oppositions such as body–mind, objective–subjective, or biomedical–traditional. In classical Aristotelian logic, if A is true, then not-A must be false (the principle of the excluded middle). In complex living systems, however, contradictory states can coexist at different levels of reality or observation (33, 35). The “third” is not a compromise or middle ground, but a distinct level where apparent opposites can both be valid. For example, water can be simultaneously liquid (at room temperature) and solid (when frozen)—not contradictory, but different states at different conditions. Applied to health, a person with controlled blood pressure (biomedical assessment) experiencing severe existential suffering (phenomenological assessment) is not a contradiction requiring us to choose one reality over the other. Both are true, and the “third” is the integrated clinical picture that holds both truths (36).

As a methodological tool, the logic of the included third provides practical guidance for transdisciplinary collaboration in health teams (34, 37). It legitimizes the coexistence of multiple explanatory frameworks without requiring one to dominate or invalidate the others. In practice, this means: a family health team can simultaneously use pharmacological treatment (biomedical), community therapy circles (psychosocial), and medicinal plants (traditional knowledge) for the same patient, recognizing that each addresses different dimensions of the chronic condition. A diagnostic assessment can integrate laboratory markers, biographical narrative, and spiritual meaning-making as complementary, not competing, sources of knowledge about the person's health state (38). Health policies can support high-technology interventions (such as CPAP devices) alongside nature-based and community-based practices, understanding them as resources acting at different levels of causation rather than as opposed paradigms (4, 6).

As a clinical decision criterion, the logic of the included third helps practitioners recognize when biomedical interventions have resolved part of a complex problem but other dimensions remain unaddressed (12). Consider the clinical vignette presented in Section 3.3: after CPAP treatment, the patient's blood pressure improves and nocturnal apnoeas disappear (biomedical success), yet chronic muscle tension, accelerated routines, and loss of life purpose persist (unchanged mental, vital and supramental (meaning and purpose) dimensions). A binary logic would frame this as either “treatment success” (blood pressure controlled) or “treatment failure” (patient still suffers). The logic of the included third recognizes both statements as simultaneously true and signals the need to expand care to include physiotherapy, psychotherapy, biographical counseling, or other resources that address the untouched dimensions.

In terms of policy, the logic of the included third invites universal systems to move beyond the polarization between “conventional medicine” and “traditional or complementary practices” (5, 6). Instead, it encourages frameworks in which different knowledge systems are brought into dialogue, provided that they converge toward more integral care, safety and equity. This does not mean uncritical acceptance of all practices, but rather the creation of regulatory, educational and organizational conditions for rigorous, respectful integration (39).

Operationally, the logic of the included third can be applied through: (1) Training health professionals to recognize and work with multiple valid perspectives on the same clinical situation (37). (2) Designing care protocols that explicitly integrate resources from different knowledge systems (e.g., antihypertensive medication + meditation + community support groups) (38, 40). (3) Developing evaluation frameworks that assess outcomes across multiple dimensions (biomedical parameters + quality of life + sense of meaning + community belonging) rather than privileging one type of outcome (3, 12). (4) Creating institutional spaces (case discussions, interprofessional meetings) where different perspectives can be articulated and integrated into coherent care plans (34, 39).

### 2.3 Integrating the pillars: from epistemology to praxis

The three pillars (complexity, multidimensionality and the logic of the included third) are not isolated concepts but form an integrated architecture that shapes how we understand and intervene in chronic conditions. This section clarifies how these pillars relate to each other and how, taken together, they lead us to recognize and work with downward causation as a key operational mechanism in transdisciplinary integrative health.

The relationship among the pillars can be understood as follows: complexity (first pillar) establishes that living systems, including human beings and health-illness processes, are characterized by non-linear causation, emergent properties and multilevel organization. This complexity perspective opens conceptual space for the second pillar, the multidimensional conception of the human being, which operationalizes complexity by identifying distinct but interconnected dimensions (physical, metabolic, vital, mental, supramental) through which chronic conditions manifest and can be addressed. The logic of the included third (third pillar) provides the epistemological and methodological bridge that allows practitioners to hold multiple perspectives (biomedical, phenomenological, traditional, ecological) without forcing them into a single reductionist framework. Rather than choosing between competing explanations at different levels, the logic of the included third legitimizes their coexistence as complementary views of the same complex reality.

Together, these three pillars create the conceptual conditions for recognizing downward causation. Downward causation refers to the capacity of higher-level patterns (meanings, narratives, relationships, social arrangements, spiritual experiences) to exert influence on lower-level processes, including biological and physiological ones (41, 42). In the context of chronic conditions, this means that changes at the supramental dimension (sense of life purpose, spiritual connection), mental dimension (beliefs, emotional tone, relational patterns) or vital dimension (daily rhythms, posture, movement quality) can influence metabolic regulation (inflammatory markers, autonomic balance, immune function) and physical manifestations (blood pressure, pain, structural changes).

Downward causation is not a separate theoretical principle independent of the three pillars; rather, it is what becomes visible and actionable when we adopt the transdisciplinary integrative perspective they define. Without complexity thinking, we would remain locked in linear models where causation flows only from genes and molecules upward. Without multidimensionality, we would lack a structured way to identify the levels at which intervention can occur. Without the logic of the included third, we might treat downward causation skeptically (as “unscientific” or incompatible with biomedical mechanisms) rather than recognizing it as complementary to upward causation (from molecules to experience).

There is a growing empirical literature consistent with downward causation in health, although it is often not framed in these terms. Research in psychoneuroimmunology demonstrates that psychosocial experiences (stress, social support, meditation practice) influence gene expression, immune cell function and inflammatory pathways (43, 44). Studies in social epidemiology show that community participation, sense of purpose and meaningful social roles are associated with reduced cardiovascular risk, slower cognitive decline and longer telomere length (45, 46). Contemplative neuroscience reveals that meditation and mindfulness training produce measurable changes in brain structure, autonomic regulation and metabolic parameters (47, 48). These findings illustrate downward causation in action: changes at higher organizational levels (meaning, relationships, contemplative practice) cascade down to influence molecular and physiological processes.

In the transdisciplinary integrative health approach, recognizing downward causation has several practical implications. First, it expands the therapeutic field: interventions that address meaning, purpose, relationships or spiritual experience are not merely “adjuncts” to biomedical treatment but legitimate components of care that can influence disease trajectories through measurable biological pathways. Second, it reframes what counts as “mechanism”: explaining how a TCIH practice works is not limited to identifying active compounds or direct biochemical effects, but can include understanding how the practice influences mental patterns, vital rhythms or supramental (meaning and purpose) awareness, which then affect metabolic and physical dimensions. Third, it suggests that effective care for chronic conditions often requires working at multiple levels simultaneously: biomedical interventions to address urgent physical or metabolic dysregulation, combined with TCIH practices, psychotherapeutic support, community engagement and nature-based interventions that act through downward causation to shift the patterns sustaining illness.

Importantly, downward causation does not replace or invalidate upward causation (the well-established pathways from molecular and cellular processes to organs, systems and experience). Both directions of influence operate simultaneously in living systems. A person's chronic pain may have clear structural causes (joint degeneration, nerve compression) that require physical interventions, while also being influenced by mental patterns (catastrophising, fear of movement) and existential meanings (pain as punishment, loss of life purpose) that can be addressed through psychological and spiritual care. The logic of the included third allows us to work with both upward and downward causation as complementary rather than competing explanations.

This integrated architecture (complexity enabling multidimensionality, both legitimized by the logic of the included third, together revealing downward causation as an operational mechanism) provides the conceptual scaffold for the clinical and policy applications discussed in subsequent sections. It clarifies why the transdisciplinary integrative health approach is not simply adding TCIH to conventional care, but fundamentally reframing chronic conditions as multilevel, bidirectional processes that require correspondingly multilevel, integrated responses.

## 3 Reframing chronic conditions in universal health systems

### 3.1 From linear risk-factor logic to complex biographical processes

Frameworks used in universal systems to address chronic conditions are usually organized around risk factors and diagnostic labels. They have been useful for identifying modifiable determinants and guiding preventive interventions, but they tend to narrow chronic conditions to a static interaction between “risk” and “disease” within the individual body. They reinforce a focus on controlling parameters and individual behavior change, often marginalizing subjective experience, structural determinants and ecological contexts.

Integrating the three pillars described above allows chronic conditions to be reframed as complex, multidimensional and relational processes. Rather than being defined only as long-lasting diseases with specific codes, chronic conditions can be described as long-term configurations of physical and metabolic alterations, vital tone, mental patterns, supramental (meaning and purpose) dynamics, social positions and ecological contexts. This invites health professionals to ask not only “How do we control this parameter?” but also “How does this condition make sense in this biography and territory?” and “What systemic conditions are sustaining or transforming this situation?”.

### 3.2 An illustrative example: arterial hypertension beyond the biomolecular model

Arterial hypertension offers a clear example of how a transdisciplinary integrative approach can complement the conventional biomolecular model. In current guidelines, hypertension is framed as a chronic disease defined by cut-off values for blood pressure and a set of cardiovascular risk factors. Clinical encounters therefore focus on pharmacological control and standardized lifestyle advice, with limited space for exploring biographical, relational or environmental dimensions.

From a transdisciplinary perspective, arterial hypertension can be understood as a pattern that expresses imbalances in physical and metabolic regulation (for example, vascular changes, inflammatory profile, autonomic activity), vital dynamics (loss of rhythm, chronic tension, posture changes), mental life (beliefs about control, fear, unresolved grief, worry about the future), and supramental (meaning and purpose) dimensions (life projects, perceived purpose, long-standing family dynamics). The practical consequence is not to abandon evidence-based pharmacological treatment, but to expand the therapeutic field: conventional biomedical care, TCIH practices, community processes and nature-based interventions become legitimate and complementary components of care.

**Figure 1:**
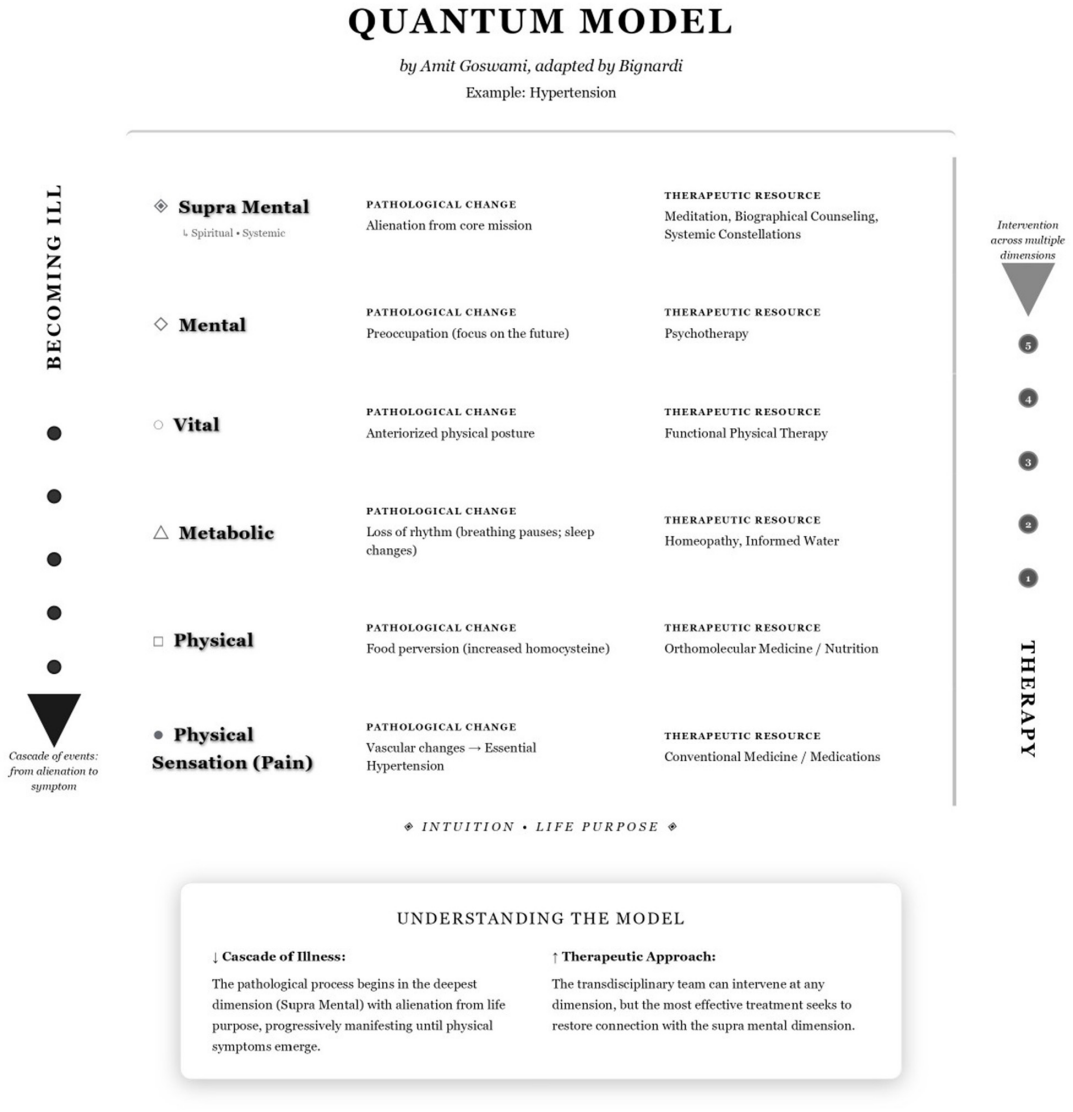
Quantum-inspired multidimensional model of chronic illness applied to arterial hypertension. The diagram organizes the human being into five dimensions of expression: supramental, mental, vital, metabolic, and physical. On the left, the vertical axis “becoming ill” represents a cascade from alienation in the supramental dimension (loss of life purpose) to physical manifestations. Physical sensation (pain) is shown at the base not as a separate sixth dimension, but as the symptomatic manifestation through which disturbances in the physical dimension become consciously experienced. On the right, the vertical axis “therapy” indicates that interventions can occur at any level, ideally supporting re-expansion toward reconnection with purpose. For each dimension, the figure lists examples of pathological change and corresponding therapeutic resources, using arterial hypertension in older adults as an illustrative case (adapted from conceptual work by Goswami and Bignardi).

A quantum-inspired multidimensional diagram summarizes this model, mapping the five dimensions, main aspects observed in each one, instruments used to explore them, and possible therapeutic resources (Figure 1).

In this framework, “disease” (dis-ease) is no longer only a number above a threshold, but a configuration in which the person's life no longer flows as it could across physical, metabolic, vital, mental and supramental (meaning and purpose) domains.

### 3.3 A concrete vignette: supradisciplinary diagnosis and the cascade of therapeutic possibilities

In conventional healthcare, each professional discipline produces its own diagnosis: the physician identifies pathophysiological alterations, the psychologist maps cognitive-emotional patterns, the physiotherapist evaluates functional limitations, and so forth. Even when these professionals work side by side, the resulting picture is typically a juxtaposition of parallel assessments, each framed by its own epistemological lens. A supradisciplinary diagnosis, by contrast, is not the sum of disciplinary parts but an emergent, integrative reading of the person as a whole. Drawing on phenomenological observation as a shared method (attending to bodily rhythms, postural patterns, narrative themes, relational dynamics, and existential orientation) the care team constructs a unified clinical portrait that cuts across disciplinary boundaries. The qualifier supra signals that this diagnostic process operates at a level of complexity that no single discipline can reach alone: it requires the simultaneous consideration of how disturbances manifest, interact and reinforce one another across the five dimensions of human expression.

The practical product of a supradisciplinary diagnosis is a dimensional cascade map: an explicit identification of how the person's condition unfolds from one dimension to another—for instance, from loss of existential meaning (supramental: meaning and purpose) through ruminative thought patterns (mental) to autonomic dysregulation (vital), metabolic imbalance, and structural deterioration (physical). Once this cascade is made visible, the therapeutic team can design interventions at each dimensional level, selecting practices from different traditions (biomedical, mind-body, contemplative, manual) according to where they are most likely to trigger reorganization through downward causation. In this way, the supradisciplinary diagnosis serves as the operational bridge between the multidimensional theoretical framework and individualized clinical action. The following vignette illustrates how this diagnostic process unfolds in practice.

*Operationally, the supradisciplinary diagnosis and dimensional cascade map follow a structured process:*

Inputs (individual assessments): each team member conducts a dimension-sensitive assessment using phenomenological observation (attending to bodily rhythms, postural patterns, narrative themes, relational dynamics, and existential orientation) alongside discipline-specific tools. The family physician contributes biomedical and metabolic data; the physiotherapist, postural and vital observations; the psychologist, cognitive-emotional patterns; the community health worker, social and territorial context; and the TCIH practitioner, energetic and contemplative dimensions.

Process (supradisciplinary synthesis): in a structured interprofessional case discussion guided by the logic of the included third, the team integrates individual assessments into a unified portrait. Rather than juxtaposing parallel reports, practitioners identify how disturbances in one dimension sustain or amplify disturbances in others. The key question is: “What is the cascade through which this person's condition is sustained across dimensions?” The team constructs a dimensional cascade map, tracing the pathways from higher-level patterns (e.g., loss of purpose) through intermediate dimensions (e.g., ruminative thought, disrupted rhythms) to physical manifestations (e.g., sustained hypertension).

Outputs (care plan): (a) A dimensional cascade map identifying the primary cascade sustaining illness and the dimensions most amenable to intervention; (b) a multidimensional therapeutic plan specifying interventions at each relevant dimension, the responsible practitioner(s), and expected interactions between interventions; and (c) re-evaluation criteria defining what changes across dimensions would signal therapeutic progress beyond biomedical parameter control.

*Clinical case:* an older adult with long-standing arterial hypertension, obesity and obstructive sleep apnoea (OSA) receives conventional treatment including antihypertensive medication and nocturnal continuous positive airway pressure (CPAP). After six months of regular CPAP use, biomedical parameters improve significantly: blood pressure decreases from 160/100 mmHg to 130/80 mmHg, nocturnal oxygen desaturations disappear, and polysomnography confirms resolution of apnoeic episodes. Clinical trials have demonstrated that CPAP can reduce blood pressure in patients with OSA and resistant hypertension (49).

From the viewpoint of the biomolecular model, the therapeutic goal appears achieved: the pathophysiological cascade from apnoea to sympathetic hyperactivation to hypertension has been interrupted. A supradisciplinary assessment, however, reveals that only part of the multidimensional pattern has changed:

*Physical dimension:* blood pressure normalized, apnoeas resolved. Chronic neck and shoulder muscle tension persists (observed through palpation, restricted cervical range of motion).

*Metabolic dimension:* improved oxygenation, reduced inflammatory markers (C-reactive protein decreased). Body mass index unchanged; dietary patterns remain centered on processed foods consumed rapidly.

*Vital dimension:* respiratory rhythm during sleep normalized. Daily life rhythms remain dysregulated: irregular meal times, fragmented sleep despite CPAP, minimal physical activity, prolonged indoor screen time disconnected from natural circadian cues.

*Mental dimension:* anteriorised, collapsed posture reflecting chronic worry persists. The patient reports ongoing rumination about family conflicts and financial insecurity. Belief system centered on helplessness: “nothing I do makes a difference.” Information environment dominated by anxiety-inducing news consumption and social isolation.

*Supramental (meaning and purpose) dimension:* no engagement with life purpose or meaningful social participation. Reports feeling “disconnected from everything” since retirement three years ago. No contemplative practice, spiritual community, or sense of biographical direction.

*Interpretation:* CPAP successfully addressed the physical and partially the metabolic dimensions by interrupting nocturnal hypoxia and reducing sympathetic overdrive. However, the vital (dysregulated daily rhythms), mental (ruminative patterns, helplessness beliefs) and supramental (existential disconnection) dimensions remain unchanged. The dimensional cascade map reveals that hypertension in this person is not merely a cardiovascular event but the physical expression of a multilevel process rooted in existential disorientation and sustained by maladaptive thought patterns and lifestyle dysregulation.

*Therapeutic implications:* a supradisciplinary approach would complement CPAP and antihypertensive medication with targeted interventions across unaddressed dimensions: body-based practices such as yoga or tai chi to release chronic muscular tension and restore postural alignment (physical); nutritional counseling attentive to the rhythm and quality of eating, not only caloric content (metabolic); structured daily routines, nature exposure and breathing practices to re-establish circadian and autonomic balance (vital); cognitive-narrative work or psychotherapy addressing helplessness beliefs and ruminative patterns (mental); and facilitated engagement with community, purposeful activity or contemplative practice to reconnect with existential meaning (supramental: meaning and purpose). The cascade map thus transforms a single biomedical diagnosis (resistant hypertension) into a multidimensional therapeutic programme in which each intervention targets a specific level while contributing to systemic reorganization through downward causation.

## 4 Implications for practice, education, policy and research

### 4.1 Clinical encounters, teamwork and territories

At the level of the clinical encounter, the reframing of chronic conditions suggests that consultations should be organized not only around measurements and prescriptions but also around narratives, meanings and relationships. Listening to stories, exploring ambivalences, and recognizing spiritual and ecological dimensions of illness become central clinical acts, alongside physical examination and diagnostic reasoning. A multidimensional transdisciplinary attitude helps practitioners to see when biomedical interventions have changed part of the pattern (for example, blood pressure or respiratory rhythm) but other dimensions (posture, emotions, purpose) remain untouched and require additional forms of care.

At the level of teamwork, the approach encourages transdisciplinary collaboration in which different professional and experiential knowledges are brought into dialogue. No single discipline is assumed to hold a complete view of complex situations; instead, the logic of the included third supports the coexistence of multiple explanations as long as they converge in caring for the person and the territory.

At the level of community and territory, chronic conditions are not seen simply as aggregates of individual cases but as phenomena rooted in social, economic, cultural and ecological configurations. Group activities, community projects and intersectoral initiatives can be recognized as components of care rather than optional complements to biomedical interventions. Nature-based practices and community therapies illustrate how reconnecting with others and with environments can influence immune function, cardiovascular parameters and stress physiology (4).

### 4.2 Education, research, governance and operationalization

For health professions education, the transdisciplinary integrative approach implies that complexity, multidimensionality and the logic of the included third should be integrated into core training. Students need opportunities to engage with real-life situations in which chronic conditions are experienced as complex biographical processes, to practice dialogue across disciplines and with communities, and to reflect critically on their own assumptions about health, disease, Nature and spirituality. Training should also cover the diverse evidence base for TCIH practices, including both promising results and uncertainties, and foster skills for critical appraisal and shared decision-making (5, 6). Pedagogical strategies may include interprofessional case-based learning, immersion in territories with high prevalence of chronic conditions, supervised practice in TCIH modalities integrated with conventional care, and reflective portfolios that encourage students to examine their own epistemological assumptions and biases (50, 51).

In terms of knowledge production, the approach highlights the importance of methodologies capable of capturing the richness of chronic condition experiences and trajectories. Qualitative studies, participatory research, mixed-methods designs and long-term case-based investigations become central to understanding how chronic conditions emerge, are lived and are transformed (52). Conceptual refinement needs to be accompanied by operational tools and evaluative strategies that are concrete enough to inform practice, including indicators that reflect changes across multiple dimensions and levels of causation. Process evaluations, realist reviews and developmental evaluation approaches may be particularly suited to assess complex interventions that integrate TCIH with conventional care in universal systems (53, 54).

For policy and governance, taking this reframing seriously would imply revisiting how chronic conditions are inscribed in guidelines, indicators and financing models. Universal systems may need to adopt indicators that go beyond biological control to include experiences, relationships and territorial processes, and to design lines of care that are less disease-centric and more centered on the person and the context. At the population level, tools such as cluster analysis can be used to identify meaningful segments that integrate biomedical, psychosocial and contextual variables. Epidemiological segmentation offers a way to translate the multidimensional view of chronic conditions into actionable groupings for planning and prioritizing interventions in universal systems (55).

From a policy perspective, adopting a transdisciplinary integrative health approach to chronic conditions offers a concrete pathway for operationalizing SDG 3 within universal systems: it links clinical effectiveness with equity, cultural adequacy and more sustainable use of technologies. By strengthening family health teams, community-based practices and nature-based interventions, it also supports SDG 10 by reducing inequities in access to integrative care and SDG 4 by calling for new educational models that prepare health professionals to work with complexity and TCIH (4–6).

Political feasibility and engaging decision-makers: advancing TCIH integration in universal health systems requires not only conceptual frameworks and implementation strategies but also pragmatic arguments and advocacy approaches capable of engaging decision-makers operating within resource-constrained environments and biomedically dominated institutional cultures. Economic arguments may prove particularly persuasive: emerging evidence suggests that integrative approaches may reduce polypharmacy, decrease emergency department utilization and hospitalisations for certain chronic conditions, and improve patient-reported outcomes at comparable or lower costs than conventional care alone. However, effects are likely condition-specific and programme-dependent. In the United Kingdom's National Health Service, social prescribing initiatives (which share conceptual ground with the territorial and multidimensional approach proposed here) have reported promising but heterogeneous evidence on health service use and cost-related outcomes, with important contextual variation across programmes (56). Similarly, in Germany and Switzerland, physician-provided complementary medicine and anthroposophic therapies have been reimbursed or included in coverage decisions in specific contexts, supported by evolving evidence on outcomes and costs, although the scope and conditions of coverage vary considerably between jurisdictions (57–59). Advocacy strategies for universal systems should include: (a) establishing rigorous pilot projects with transparent evaluation of both clinical and economic outcomes, ensuring credibility with skeptical policymakers; (b) identifying and supporting institutional champions (clinicians, managers or policymakers who recognize the limitations of parameter-focused care and are willing to experiment with integrated approaches); (c) forming strategic partnerships between academic institutions and health services to conduct implementation research and generate locally relevant evidence; (d) documenting patient and community testimonials alongside quantitative data, as experiential accounts can complement biomedical evidence in making the case for more holistic care; (e) aligning integration efforts with broader policy priorities such as SDG achievement, planetary health agendas, reduction of health inequities, and primary care strengthening. To overcome cultural and institutional resistance, universal systems may need: purposeful leadership that models epistemological openness and values diverse forms of knowledge; career incentives and recognition for practitioners engaging in interprofessional collaboration and TCIH integration; dedicated time and protected spaces for dialogue across professional and epistemic boundaries; and transparent communication about both the promise and the uncertainties of integrative approaches, avoiding either uncritical enthusiasm or dismissive rejection. The Brazilian SUS's National Policy on Integrative and Complementary Practices (PNPIC), despite implementation challenges, represents one international example of institutional commitment to TCIH within a universal public system; other universal systems might draw lessons from both its successes (political legitimacy, breadth of practices recognized) and its ongoing challenges (uneven territorial distribution, variable quality assurance, insufficient training infrastructure).

Implementation pathways and risk mitigation strategies: the operationalization of this approach in universal health systems such as the SUS requires careful attention to implementation strategies and potential risks. We propose a phased, territory-based approach that begins with pilot experiences and progressively expands based on evaluation findings.

How to test the approach: (1) Pilot projects in selected territories: municipalities or health districts with existing PNPIC infrastructure and committed family health teams can serve as initial testing grounds. Pilots should include: (a) training of at least two family health teams in the transdisciplinary framework and multidimensional assessment; (b) co-design of care pathways integrating conventional, TCIH and community resources for 2–3 prevalent chronic conditions (e.g., arterial hypertension, diabetes, chronic pain); (c) establishment of regular interprofessional case discussion sessions using the logic of the included third; (d) mixed-methods evaluation over 12–18 months assessing clinical, experiential, relational and territorial outcomes. (2) Interprofessional training programmes: regional health schools or universities can develop short courses (40–60 hours) for family health teams, combining theoretical modules (complexity, multidimensionality, epistemology of TCIH) with practical immersion (supervised practice in TCIH modalities, community mapping, biographical interview techniques). Training should be accompanied by mentorship and communities of practice to support sustained implementation (51, 60). (3) Participatory action research: involving health workers, users, community leaders and TCIH practitioners in the co-production of knowledge about what works, for whom, and under which conditions. This approach can generate locally adapted protocols while maintaining fidelity to core principles (61).

At which levels of the system: primary care (priority level): Family health teams are the strategic entry point. The transdisciplinary integrative approach should be embedded in everyday clinical encounters, home visits, group activities and community engagement, not relegated to specialist referrals. Network level (municipalities/health regions): Coordination between primary care, NASF teams, integrative practices centers, mental health services (CAPS), and community resources. Shared care plans, interprofessional meetings and integrated information systems are essential to avoid fragmentation. State and national levels: development of clinical guidelines that incorporate multidimensional assessment, financing mechanisms that support time for narrative-based consultations and interprofessional work, regulation and quality assurance for TCIH practices, and inclusion of transdisciplinary competencies in accreditation standards for health professions education (15, 16).

Mitigating potential risks: risk 1: fragmentation of care. TCIH services may operate in parallel to conventional care without genuine integration, creating duplication, conflicting messages or gaps. Mitigation strategies: (a) Shared electronic health records accessible to all team members; (b) mandatory interprofessional case discussions for complex chronic conditions; (c) single care coordinator for each patient with multimorbidity; (d) integration of TCIH competencies into family physician and nurse training rather than creating separate specialist roles (11, 21). Risk 2: inequalities in access. TCIH may become a “luxury” for urban, educated populations or conversely a “second-class” option for vulnerable groups when high-technology care is unavailable. Mitigation strategies: (a) Explicit equity criteria in service planning (e.g., prioritize territories with high burden of chronic conditions and limited specialist access); (b) free provision within SUS to avoid market-based barriers; (c) culturally adapted TCIH practices co-designed with local communities, including indigenous and traditional populations; (d) monitoring of utilization patterns disaggregated by income, race/ethnicity, and geography (10, 17). Risk 3: lack of regulation and safety concerns. Without adequate oversight, unsafe practices, unqualified practitioners or exploitation of traditional knowledge may occur. Mitigation strategies: (a) Clear accreditation requirements for TCIH practitioners working in SUS, including both technical competence and ethical training; (b) Informed consent protocols that explain what is and is not evidence-based; (c) Adverse event monitoring systems; (d) Respectful protection of traditional knowledge through community-based governance and benefit-sharing agreements; (e) Continuous professional development and peer review (9, 15). Risk 4: dilution of the transdisciplinary approach. The framework may be reduced to simply “adding” acupuncture or meditation to existing services without genuine epistemological dialogue or transformation of practice. Mitigation strategies: (a) Make the three pillars (complexity, multidimensionality, logic of the included third) explicit in all training materials and protocols; (b) Evaluate not only clinical outcomes but also process indicators reflecting transdisciplinary collaboration (e.g., frequency of interprofessional meetings, use of multidimensional assessment tools, patient-reported experience of integrated care); (c) Leadership commitment and institutional culture change, not just technical innovation (34, 37, 51).

### 4.3 Training family health teams for transdisciplinary integrative practice

In systems such as the SUS, family health teams (comprising family physicians, nurses, nursing technicians and community health workers, often supported by NASF (Family Health Support Center) professionals) are strategically positioned to operationalize transdisciplinary integrative approaches to chronic conditions. This requires both individual competencies and organizational conditions that support integrated practice.

Training programmes for transdisciplinary practice:

To equip teams for this approach, training should include:

(1) Conceptual foundations: understanding complexity, multidimensionality and logic of the included third through case-based learning, reflective exercises and dialogue with lived experiences of chronic conditions (50, 51). This includes recognizing how changes at multiple levels (meanings, relationships, community participation) can influence physiological processes and disease trajectories (41–44).
(2) Assessment competencies: training in multidimensional anamnesis that extends beyond biomedical history to inquire about vital rhythms, explore biographical narratives, attend to posture and movement, and create safe space for discussing existential and spiritual dimensions (62).
(3) Interprofessional collaboration skills: structured case discussion formats using the logic of the included third; shared decision-making protocols that integrate perspectives from different disciplines and knowledge systems; conflict resolution strategies when epistemological differences arise (51, 60).
(4) TCIH literacy: basic understanding of evidence, mechanisms and appropriate use of TCIH practices commonly available in SUS (acupuncture, herbal medicine, meditation, yoga, community therapy). Not all team members need to practice all modalities, but all should understand their rationale and be able to guide patients appropriately (17, 18).
(5) Community engagement and territorial diagnosis: methods for participatory mapping of community resources, identification of local healing traditions, co-design of care pathways with community members, and recognition of nature-based resources as health assets (61, 63).

Organizational practices that enable integrated care:

Beyond individual training, institutional arrangements must support transdisciplinary practice:

Protected time for comprehensive consultations: financing and scheduling models that allow 30–40 min appointments for complex chronic conditions, enabling exploration of multiple dimensions rather than rushed problem-focused encounters (3, 64).

Dedicated spaces for group and contemplative practices: physical spaces in health units for meditation circles, therapeutic movement, community therapy, arts-based practices—recognized as core services, not peripheral activities (20).

Care plans connected to life purpose and community: instead of focusing exclusively on parameter control and behavior change, care plans that explicitly ask “What gives this person's life meaning?” and “What community connections can be strengthened or rebuilt?” These questions open therapeutic pathways that address mental and supramental (meaning and purpose) dimensions while influencing metabolic and physical processes (38, 62).

Integration of nature-based practices: formal incorporation of prescriptions for nature contact (e.g., “30 min daily walk in the park”, “weekly participation in community garden”), potentially in partnership with environmental and education sectors. This operationalizes planetary health perspectives while creating conditions for multilevel therapeutic effects (4, 63).

Regular interprofessional case discussions: weekly or biweekly team meetings using structured protocols for multidimensional case review. Cases are presented through narratives that include vital, mental and supramental (meaning and purpose) dimensions, not solely through biomedical parameters. Different team members contribute perspectives; the logic of the included third legitimizes apparent contradictions as complementary views rather than competing truths (34, 51).

Institutional culture of epistemological openness: leadership that models openness to different knowledge systems, acknowledges uncertainty, and prioritizes learning over rigid protocol adherence. This cultural shift enables TCIH to be integrated as genuine dialogue partners rather than subordinated “alternatives” (37, 39).

From individual care to territorial transformation:

When family health teams work through this framework (attending to multiple dimensions, integrating TCIH with conventional care, and connecting individual care plans to community and ecological contexts) they contribute not only to individual healing but to territorial transformation. Teams become agents of change extending beyond clinic walls: fostering community solidarity, reconnecting people with Nature, creating spaces for meaning-making, and challenging structural conditions that generate chronic suffering (13, 14, 63). In this sense, TCIH is not an add-on but a set of tools and attitudes that help teams respond to chronic conditions in ways more coherent with people's lived experiences, more aligned with ecological limits, and more conducive to sustainable universal health systems (4–6).

## 5 Scope and conceptual boundaries of the proposed framework

The five-dimensional model (physical, metabolic, vital, mental, supramental) is heuristic rather than empirically validated. We have not demonstrated that these five dimensions represent distinct, measurable constructs, nor that they exhaust the relevant aspects of human experience in chronic illness. The model serves as an organizing device to expand clinical attention beyond biomedical parameters, but it should not be reified as ontological truth. Alternative dimensional schemas (such as the four-dimensional model used in some anthroposophic medicine traditions or the three-dimensional frameworks in certain indigenous healing systems) may be equally valid or more appropriate in specific contexts.

The “supramental dimension” warrants particular scrutiny. This term (encompassing both spiritual and systemic aspects) refers to transpersonal awareness and contemplative experience (spiritual) as well as biographical configurations and participation in larger patterns of meaning and destiny (systemic). While we find this dual framing conceptually valuable for capturing dimensions of meaning, purpose and transcendence that influence health trajectories, we acknowledge that it is not standard terminology in English-language public health literature and may be perceived as vague, culturally specific, or inappropriately importing spiritual language into secular health systems. Different cultural traditions conceptualize spirituality, meaning-making and connection to larger patterns in distinct ways; our framing reflects specific philosophical influences (particularly integral theory and transdisciplinary epistemology) (31, 32) that are not universally shared. Healthcare systems and practitioners from other traditions may need different language to engage these dimensions meaningfully.

Operationalising the logic of the included third remains challenging. While we describe it as simultaneously an epistemological principle, methodological tool and clinical decision criterion (Section 2.2), we have not provided validated protocols, decision algorithms or assessment instruments that would enable practitioners to apply it consistently. The risk is that “logic of the included third” becomes a rhetorical device for legitimizing contradictions without rigorous analysis of when genuine complementarity exists vs. when epistemological differences reflect irreconcilable commitments. Future work must develop concrete methodologies (case discussion protocols, decision support tools, quality criteria for transdisciplinary integration) that prevent the concept from collapsing into uncritical eclecticism.

The relationship between the three pillars (complexity, multidimensionality, logic of the included third) and the mechanism of downward causation requires further clarification. Is downward causation a consequence of the logic of the included third, an independent explanatory principle, or an empirical phenomenon that the three pillars help us recognize? The manuscript implies but does not explicitly articulate this architecture, creating potential conceptual confusion. A more rigorous exposition would specify the logical dependencies among these concepts and empirically demonstrate their utility for understanding chronic conditions.

## 6 Concluding remarks

Chronic conditions pose one of the greatest challenges to universal health systems, not only because of their prevalence and costs, but because they expose the limits of reductionist ways of understanding health and disease (1–3). When chronic conditions are treated mainly as static diagnostic labels and as the end result of linear risk-factor chains, systems tend to respond with fragmented interventions that focus on parameter control and individual responsibility, while leaving untouched many of the social, ecological and existential roots of suffering.

This Perspective makes three principal contributions to the literature on Traditional, Complementary and Integrative Health (TCIH) within universal health systems. First, conceptually, it provides an explicit architectural integration of complexity theory, multidimensionality (physical, metabolic, vital, mental and supramental (spiritual and systemic) dimensions), and the logic of the included third, demonstrating how these three pillars together reveal downward causation as an operational mechanism through which interventions at mental, vital or supramental levels can influence metabolic and physical processes. Second, methodologically, it offers a five-dimensional assessment framework and structured protocols (interprofessional case discussions, multidimensional anamnesis, territorial diagnosis) that operationalize transdisciplinary principles for clinical practice, moving beyond abstract calls for “integration” to concrete tools that practitioners can use. Third, practically and politically, it outlines feasible implementation pathways for training family health teams, organizing services and engaging decision-makers in resource-constrained universal systems, drawing on international examples (NHS social prescribing, German-Swiss anthroposophic integration, Brazilian PNPIC) to demonstrate that epistemological plurality and TCIH integration are achievable within publicly funded health systems when supported by pragmatic economic arguments, rigorous pilot projects and strategies for cultural change.

This Transdisciplinary Integrative Health Approach reframes chronic conditions in universal systems not by opposing the biomedical model but by expanding and complementing it. Grounded in the pillars of complexity, multidimensionality and the logic of the included third, this approach invites chronic conditions to be seen as complex, biographical and relational processes rather than static diagnostic labels. It creates space for dialogue among different forms of knowledge and care (conventional biomedical interventions, TCIH practices, community-based resources and nature-based approaches) positioning them as complementary resources acting at different dimensions and levels of causation (4–6).

Its value will depend on the capacity of practitioners, educators, policymakers and communities to translate these concepts into concrete experiments, tools and institutional innovations, and to evaluate their effects. Future work may explore experiences that embody elements of this approach in different settings; develop instruments for assessment, planning and reflection consistent with its three pillars and five dimensions; and dialogue with existing frameworks such as chronic care models, social determinants of health, planetary health, lifestyle medicine and syndemic perspectives (3, 4). Engaging with these paths may help universal health systems, including the SUS, to care more integrally for people living with chronic conditions in a world marked by crisis, but also by possibilities for reconnection and transformation.

Key future research directions include: developing multidimensional assessment tools valid for routine practice; investigating mechanisms through which TCIH practices influence different dimensions of chronic conditions; conducting implementation studies to identify effective strategies for training and organizing transdisciplinary teams; evaluating comparative effectiveness and cost-effectiveness through pragmatic trials; monitoring equity and access patterns across social determinants; and comparing implementation across diverse universal health systems to identify generalizable principles and context-specific adaptations.

## 7 Limitations

This Perspective offers a conceptual and methodological framework for integrating Traditional, Complementary and Integrative Health (TCIH) into universal health systems' responses to chronic conditions. As a theoretical proposal rather than an empirical study, it carries inherent limitations that require explicit acknowledgment. The conceptual boundaries and scope of the proposed framework (including the heuristic nature of the five-dimensional model, the non-standard terminology, and the relationship between the three pillars and downward causation) are discussed separately in Section 5. The present section focuses on limitations of the manuscript itself, the evidence base for TCIH, and implementation uncertainties and risks.

### 7.1 Limitations of the article itself

This manuscript presents a normative and heuristic framework without accompanying empirical data. We have not conducted systematic reviews of TCIH effectiveness, implementation studies in the Brazilian SUS, or controlled evaluations of the proposed transdisciplinary approach. The illustrative clinical vignette (Section 3.3) serves a pedagogical purpose but does not constitute evidence of the framework's effectiveness or feasibility. Readers should therefore understand this Perspective as a conceptual proposal intended to stimulate debate, guide future research and inform experimental implementation—not as demonstrated evidence of what works.

The framework draws primarily from the Brazilian SUS context, which may limit its generalizability. While universal health systems share common features (tax-funded coverage, public provision of services, equity commitments) they operate within diverse political, cultural and economic contexts. Implementation pathways described in Section 4 reflect Brazilian institutional structures (family health teams, NASF, PNPIC) that may not exist elsewhere. Adaptations would be necessary for other settings, and we cannot assume that insights from Brazil automatically transfer to other universal systems.

Our engagement with critical literature remains limited. Although we acknowledge controversies surrounding TCIH integration (Section 1), we have not comprehensively reviewed critiques of complexity theory, transdisciplinarity, or integrative medicine from philosophical, sociological or clinical perspectives. The manuscript's argumentative structure privileges perspectives supportive of TCIH integration, potentially creating confirmation bias. Future work should engage more deeply with dissenting voices, including those who argue for clearer boundaries between evidence-based medicine and traditional practices, those who question whether complexity theory adds explanatory value beyond existing biopsychosocial frameworks, and those who raise concerns about cultural appropriation and commercialization of indigenous knowledge.

### 7.2 Limitations of the evidence base for TCIH

Evidence quality for TCIH practices varies considerably across modalities and conditions. While some interventions (such as acupuncture for certain pain conditions, mindfulness-based stress reduction for depression and anxiety, and specific herbal medicines for defined indications) have support from rigorous trials and systematic reviews, many other practices commonly included under the TCIH umbrella have limited evidence or contradictory findings (7, 8). Heterogeneity in intervention definitions (what exactly constitutes “yoga” or “traditional medicine”?), outcome measures, comparators and populations makes synthesis difficult. Publication bias, small sample sizes, inadequate blinding and inconsistent reporting further compromise evidence certainty for numerous TCIH modalities.

Our framework does not resolve these evidential challenges. By proposing that TCIH practices can act at different dimensions and levels of organization, we risk creating a theoretical justification for continuing practices even when rigorous evaluation shows null or harmful effects. The logic of the included third must not become a license to ignore evidence or to privilege practitioner intuition over systematic inquiry. Maintaining scientific rigor while remaining open to epistemological plurality is an ongoing tension that this manuscript identifies but does not fully resolve.

Furthermore, much TCIH evidence comes from contexts very different from universal public health systems. Trials conducted in research settings with highly selected populations, extensive monitoring and specialized practitioners may not reflect real-world effectiveness when practices are delivered in under-resourced primary care settings by professionals with basic rather than expert training. Implementation science questions (who delivers which TCIH practices, to whom, under what conditions, with what training and support) remain largely unanswered for the Brazilian SUS and most other universal systems.

### 7.3 Implementation uncertainties and risks

The implementation pathways described in Section 4 are provisional and largely untested. Several risks warrant acknowledgment: fragmentation of care if TCIH services operate in parallel without genuine integration; inequalities in access if TCIH becomes available primarily to affluent populations or conversely offered as a lesser alternative when high-technology care is unavailable; insufficient regulatory oversight leading to patient harm or erosion of trust; and resource allocation tensions when TCIH competes with other priorities under fiscal constraint. Concrete mitigation strategies for each of these risks are detailed in Section 4.2. We have not conducted economic evaluations demonstrating that the proposed approach represents good value for money, and arguments for integration must eventually be reconciled with budgetary realities.

### 7.4 Concluding reflections

Acknowledging these limitations reflects the epistemic humility appropriate to complex, contested phenomena. This Perspective proposes a conceptual framework grounded in careful theoretical work while explicitly recognizing evidential gaps and implementation uncertainties. Its value will be determined not by whether it is correct in some absolute sense, but by whether it proves useful for stimulating productive inquiry, guiding thoughtful experiments, and contributing to more effective, equitable and humane care for chronic conditions within universal health systems.

## Data Availability

The original contributions presented in the study are included in the article/supplementary material, further inquiries can be directed to the corresponding author.
